# Virtual Flow-T Stenting for Two Patient-Specific Bifurcation Aneurysms

**DOI:** 10.3389/fneur.2021.726980

**Published:** 2021-11-03

**Authors:** Mengzhe Lyu, Yiannis Ventikos, Thomas W. Peach, Levansri Makalanda, Pervinder Bhogal

**Affiliations:** ^1^Department of Mechanical Engineering, University College London (UCL), London, United Kingdom; ^2^School of Life Science, Beijing Institute of Technology, Beijing, China; ^3^Department of Interventional Neuroradiology, The Royal London Hospital, London, United Kingdom

**Keywords:** T-stenting technique (Flow-T), virtual fast deployment algorithm, hemodynamic simulation, inflow reduction, wide necked cerebral aneurysms

## Abstract

The effective treatment of wide necked cerebral aneurysms located at vessel bifurcations (WNBAs) remains a significant challenge. Such aneurysm geometries have typically been approached with Y or T stenting configurations of stents and/or flow diverters, often with the addition of endovascular coils. In this study, two WNBAs were virtually treated by a novel T-stenting technique (Flow-T) with a number of braided stents and flow-diverter devices. Multiple possible device deployment configurations with varying device compression levels were tested, using fast-deployment algorithms, before a steady state computational hemodynamic simulation was conducted to examine the efficacy and performance of each scenario. The virtual fast deployment algorithm based on a linear and torsional spring analogy is used to accurately deploy nine stents in two WNBAs geometries. The devices expand from the distal to proximal side of the devices with respect to aneurysm sac. In the WNBAs modelled, all configurations of Flow-T device placement were shown to reduce factors linked with increased aneurysm rupture risk including aneurysm inflow jets and high aneurysm velocity, along with areas of flow impingement and elevated wall shear stress (WSS). The relative position of the flow-diverting device in the secondary daughter vessel in the Flow-T approach was found to have a negligible effect on overall effectiveness of the procedure in the two geometries considered. The level of interventionalist-applied compression in the braised stent that forms the other arm of the Flow-T approach was shown to impact the aneurysm inflow reduction and aneurysm flow pattern more substantially. In the Flow-T approach the relative position of the secondary daughter vessel flow-diverter device (the SVB) was found to have a negligible effect on inflow reduction, aneurysm flow pattern, or WSS distribution in both aneurysm geometries. This suggests that the device placement in this vessel may be of secondary importance. By contrast, substantially more variation in inflow reduction and aneurysm flow pattern was seen due to variations in braided stent (LVIS EVO or Baby Leo) compression at the aneurysm neck. As such we conclude that the success of a Flow-T procedure is primarily dictated by the level of compression that the interventionalist applies to the braided stent. Similar computationally predicted outcomes for both aneurysm geometries studied suggest that adjunct coiling approach taken in the clinical intervention of the second geometry may have been unnecessary for successful aneurysm isolation. Finally, the computational modelling framework proposed offers an effective planning platform for complex endovascular techniques, such as Flow-T, where the scope of device choice and combination is large and selecting the best strategy and device combination from several candidates is vital.

## Introduction

Most cerebral aneurysms preferentially occur at vessel bifurcations (1, 2). With the major advancements in endovascular treatment over the last two decades, various of treatment strategies along with dedicated devices have been developed to deal with bifurcation aneurysms. When it comes to treating more difficult Wide neck bifurcation aneurysms (WNBAs), devices such as the pCONus and pCANvas devices (Phenox, Bochum, Germany), the Pulserider (Pulsar Vascular, Los Gatos, California, USA), and the eCLIPs devices (Evasc Medical Systems Corp.) are used to cover the neck of aneurysm and assist aneurysm coiling. Alternatively, devices such as the WEB (Microvention, Aliso Viejo, California, USA) and Luna/Artisse (Medtronic, Dublin, Ireland) are utilised to disturb intrasaccular flow and are deployed within the aneurysm dome (3).

Stent-assisted coiling has shown good clinical results in the treatment of WNBAs (4–6). During the treatment of WNBAs, a mechanical scaffold is provided by stents to stabilise the coils and prevent prolapse into the parent artery. To deal with geometrically complex bifurcation aneurysms involving both daughter branches of bifurcation, a single stent may not be sufficient (7, 8). As a result, implanting double stents in different configurations such as Y or T stenting are considered frequently as endovascular treatment of WNBAs (9–11). The conventional T-stenting technique, described as non-overlapping Y-stent technique originally, is a successful method that proven to stabilise aneurysm occlusion progressively (12, 13). Y-stenting is another eligible but challenging techniques to treat WNBAs (14), this approach uses large profile microcatheters to deliver stents which makes manipulate during navigation through sharply angled side branches difficult to operate. With the development of new dedicated low-profile devices such as the Baby Leo and LVIS EVO that can be deployed through low-profile 0.17 in microcatheters, Flow-T stenting technique merged as an advancement approach based on the conventional T-stenting (13).

In the treatment of WNBAs, the clinical use of different devices along with their detailed deployment strategy remains empirical and is amenable to optimisation. There is series of vital factors needed to be considered, such as the foreshortening of FDs after placement in the aneurysm neck, which may allow coil to prolapse into the parent vessel (15, 16), the local haemodynamic environment before and after endovascular treatment is complex (17, 18), the effect on the haemodynamics inside the aneurysm by selecting different FDs (19). In the Flow-T approach, whether coiling is necessary or not is left to be proven. Clearly, the response of WNBAs following treatment by FD is understudied. Therefore, patient-specific computational fluid dynamics (CFD) models can be utilised to evaluate the effectiveness of FD treatment in WNBAs (20).

In this study, two WNBAs were virtually treated by a novel T-stenting technique (Flow-T) with Silk Vista Baby, Baby Leo and LVIS EVO devices. Multiple possible device deployment configurations were tested, using fast-deployment algorithms, before a steady state computational hemodynamic simulations were conducted to examine the efficacy and performance of each scenario.

## Materials and Methods

### Aneurysm Geometries and Clinical Approach

**Figure 1:**
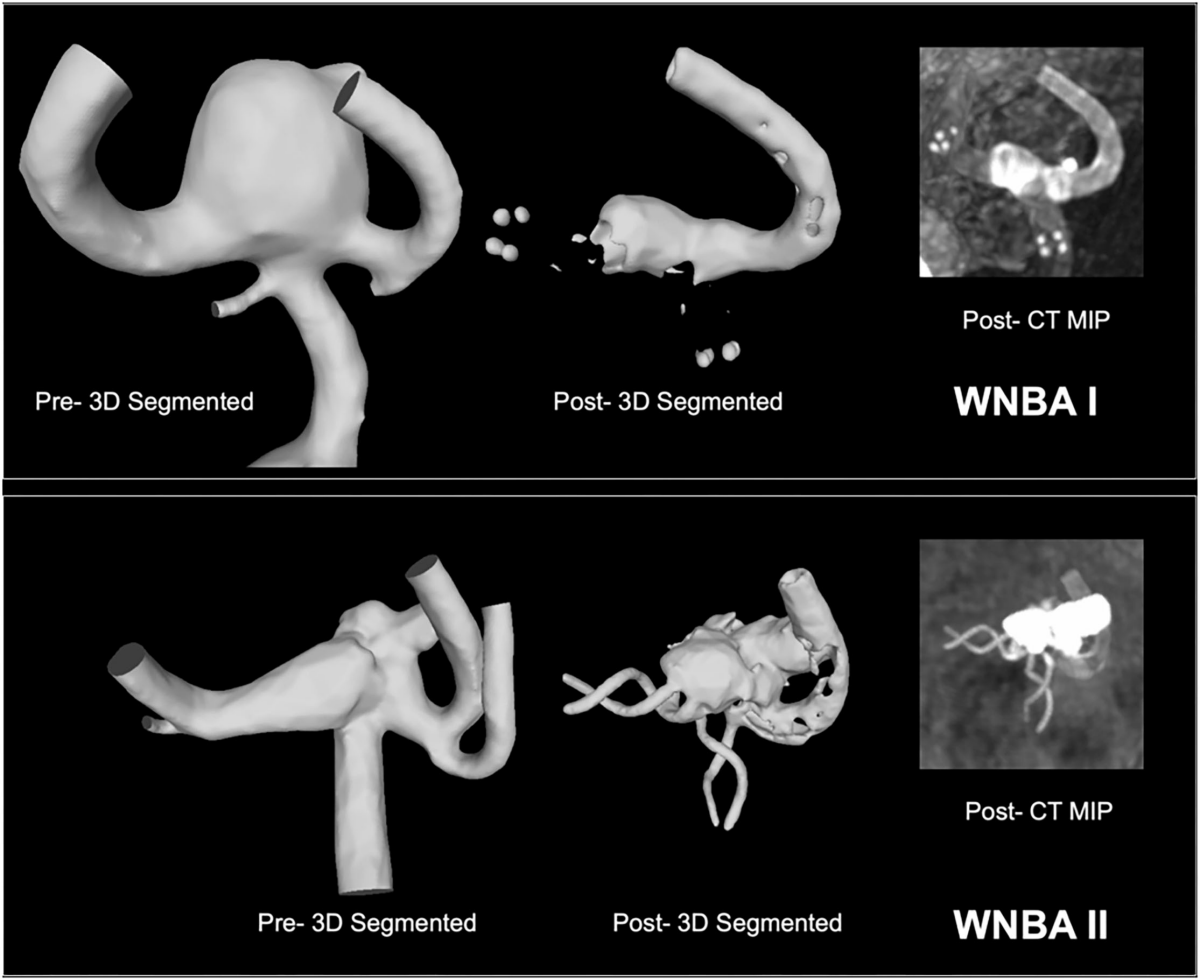
WNBA geometries I and II reconstructed from CT angiography pre-intervention with Flow-T for virtual stenting (left) and post-intervention for reference (right). Flow diverters placed in the secondary daughter vessel (right-hand in both orientations) are fully visible while the stent placed in the primary daughter vessel is indicated with end (LVIS EVO) or helical (Baby Leo) markers.

Two WNBA geometries located on the Middle Cerebral artery (MCA) that were identified for treatment were segmented from CT angiography imaging data in OsiriX (OsiriX v.4.1.1, Freeware) before being imported into Blender (Blender Foundation, Amsterdam, The Netherlands) as stereolithography (STL) format. As seen in Figure 1, the pre-intervention geometry was trimmed to produce vessel lengths of about six vessel diameters distal and proximal to the aneurysm site. Both aneurysm geometries were treated using Flow-T stenting, with the post-implantation 3D geometry and maximum intensity project (MIP) also shown in Figure 1 for reference. In the clinical approach the WNBA I case was treated with a 3 × 24 mm LVIS EVO device deployed in the primary daughter vessel (left-hand in the figure) and a 2.25 × 15 mm Silk Vista Baby flow diverter in the secondary daughter vessel. In the WNBA II case a 2.5 × 25 mm Baby Leo device was deployed in the primary daughter vessel (also left-hand in the figure) and a 2.25 × 15 mm Silk Vista Baby flow diverter in the secondary daughter vessel.

### Virtual Deployment

**Figure 2:**
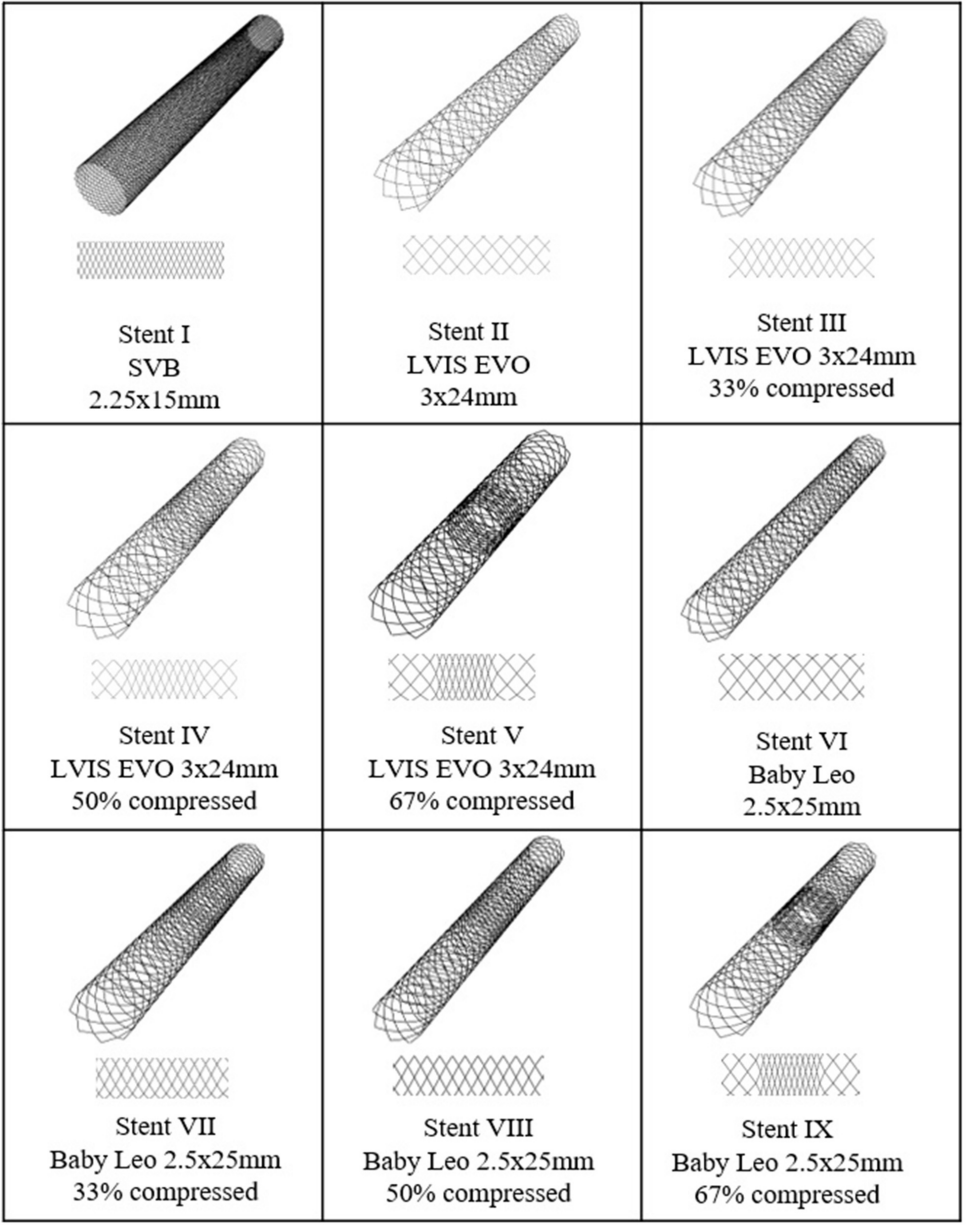
SVB, LVIS EVO, and BABY LEO designs to be virtually deployed in each WNBA geometry. Stent I is flow-diverting stent whereas stent II–VIII are low-profile braided stents with different compression level at mid third part of the devices.

In order to model the Flow-T approach virtually the three different types of devices used were reconstructed in simplified form, as shown in Figure 2. In total nine variants of the devices were created to quantify the effect of slightly different approaches to the procedure. Stent I mimics the SVB device (2.25 × 15 mm) deployed as per manufacturer instructions with a free expansion of up to 2.5 mm in diameter. Stents II-IX mimic both of the devices deployed in the primary daughter vessel with varying degrees of compression applied to the device, as these devices are compressed at the aneurysm neck during deployment to improve flow-diverting capacity. In both cases the middle third of the device (~8 mm length) can be compressed; Stents II-V represent the LVIS EVO device compressed in length by 0, 33, 50, and 67%, respectively, with an unconstrained diameter of 3.8 mm. Stents VI-IX represent the Baby Leo device compressed by length to the same degrees, with an unconstrained dimeter of 3.2 mm. Visual inspection of the post-intervention imaging confirmed in both clinical cases that the deployed configuration of the primary daughter vessel stent was most consistent with the 50% compression devices (Stent IV and Stent VIII for WNBA I and II, respectively).

The virtual deployment of devices is achieved using a fast-deployment algorithm implemented in Visual Studio 2019 (Microsoft, Albuquerque, New Mexico, USA) and Blender. Details of deployment algorithms were previously reported by the authors (21, 22). In summary, the mechanical system is discrete to a system with fictitious masses linked with springs, then the movement of such mechanical system obeys the equations of dynamic equilibrium. The contact between stent and vessel is defined to occur when the distance between the vertex of the vessel and any of the stent's vertices becomes smaller than a chosen parameter α. After contact is detected, the vertex displacements in contact are still calculated in future iterations. However, its position can only change if it is located within the α-boundary of the vessel inner surface. A wireframe representation of the device is compressed to reduce the radius and then aligned with the vessel centreline to simulate the sheltering of the device by the catheter. The unsheathing of devices is achieved by the relaxing of the device along its length progressively. The device expands to its stress-free shape (unconstrained diameter) within the limit of vessel wall. The three-dimensional device is created by adding thickness to the deployed device's wireframe, before being trimmed by removing the lengths in the parent and daughter vessels to improve the efficiency of CFD mesh generation subsequently. Device sizing, porosity and pore density is shown in Table 1 as per the for the SVB, LVIS EVO, and Baby Leo as per manufacturers' guidance (23).

**Table 1:** Stent porosity and construction.

	**Device**	**Compression [%]**	**Typical Porosity (compressed) [%]**	**Typical Pore Density (Compressed) [mm^**−1**^]**
Stent I	SVB	0	60 (–)	45 (–)
Stent II	LVIS EVO	0	70 (–)	2 (–)
Stent III	LVIS EVO	33	70 (65)	2 (3)
Stent IV	LVIS EVO	50	70 (55)	2 (4)
Stent V	LVIS EVO	67	70 (35)	2 (5)
Stent VI	Baby Leo	0	80 (–)	4 (–)
Stent VII	Baby Leo	33	80 (70)	4 (5)
Stent VIII	Baby Leo	50	80 (65)	4 (6)
Stent IX	Baby Leo	67	80 (50)	4 (10)

In addition to modelling the over- and under-compression of the stent placed in the primary daughter vessel, the relative position of the SVB flow-diverter deployed in the secondary daughter vessel was also varied. Three configurations of SVB were considered: the realistic positioning of the device inferred from visual inspection of the post-intervention angiography; an idealised positioning of the device where the SVB perfectly abuts the LVIS EVO or Baby Leo device creating a connected “T”; and finally, a poorly positioned device where a substantial gap between the SVB and primary daughter vessel device is present. These configurations are referred to generally as “real,” “ideal,” and “poor.”

### CFD Methodology

The aneurysm geometries with and without devices deployed were meshed using CFD-VisCART (ESI Group, Paris, France) using a projected single domain non-conforming unstructured mesh, an Omnitree Cartesian tree type and three near-wall Cartesian layers to give a smooth and well-resolved boundary definition. The meshes were then imported into the multi physics suite CFD-ACE+ (ESI Group) and solved assuming steady flow conditions.

Blood was modelled as an incompressible fluid with steady 3D Navier–Stokes governing equations that were solved following the finite volume approach, with a central differencing scheme for spatial interpolations. The SIMPLE Consistent (SIMPLEC) pressure correction method (24, 25) and an algebraic multigrid method for convergence acceleration (26) were used. Given previous studies in the literature that confirmed the non-Newtonian effects of blood to be small in the cerebral circulation, (27, 28) blood is modelled as a Newtonian fluid with a density of 1,000 kg/m^3^ and a dynamic viscosity of 0.004 Pa s. Arterial walls were modelled as rigid, with the effect of such an assumption on flow patterns having been shown to be negligible (29). A no-slip boundary condition was imposed on both the vessel walls and device struts.

A radially symmetric inlet velocity boundary condition was applied to each geometry scaling the corresponding velocity to a mean internal carotid artery (ICA) flow rate of 230 ml/min. A fixed pressure outlet boundary condition was applied to all geometry outlets; more complex outflow conditions incorporating Windkessel models were considered but rejected given very little variation in daughter vessel flowrates under a constant pressure condition when compared to physiological values (to within 5% of mean flow rates reported in the literature). Convergence criteria of absolute or relative residual reduction to 1 × 10^−8^ and 1 × 10^−5^ were employed.

Mesh independence to within 2% for both aneurysm inflow and wall shear stress (WSS) magnitude was assumed by meshing the geometries with a mesh density >5,000 elements per mm^3^, as per similar studies by the authors (30, 31). This resulted in mesh sizes of 4.69–10.7 million elements across the cases. CFD simulation results are post processed by visualising the WSS distributions and velocity streamlines withing the aneurysm dome. A neck plane defining the boundary of the aneurysm is place proximal to the deployed devices and allows the inflow into the aneurysm to be monitored.

## Results and Discussion

### Virtual Deployment

**Figure 3:**
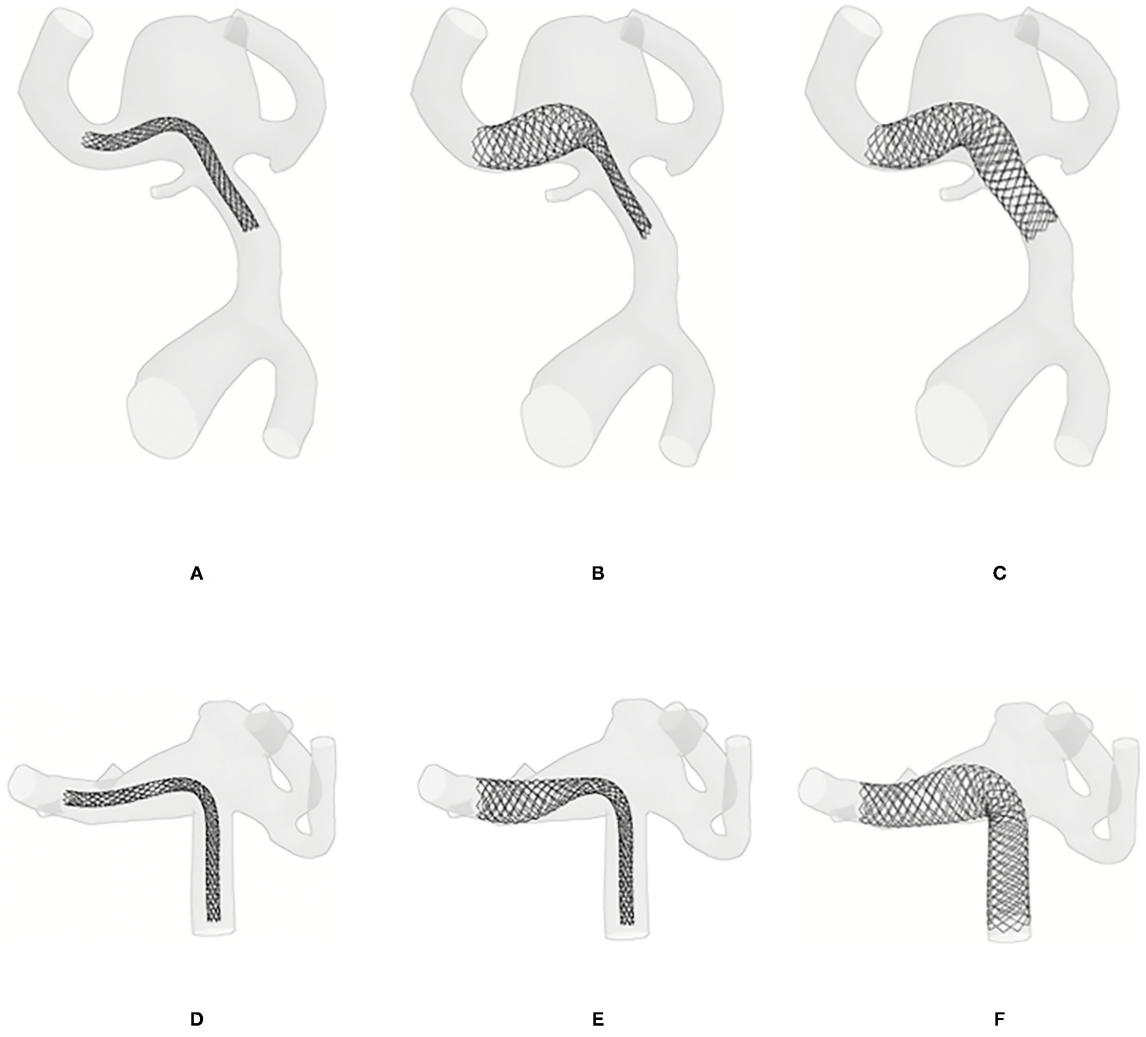
Virtual deployment process. **(A)** Crimped LVIS EVO flow-diverter placed on the centerline of the target vessel. **(B)** Half way through the LVIS EVO expansion process, starting from the distal to proximal side of the device with respect to aneurysm sac. **(C)** Configuration of full expanded LVIS EVO. **(D–F)** The deployment of BABY LEO in WNBA II.

The fast virtual deployment algorithm was applied to each device (Stents I-IX) in two WNBA geometries as shown in Table 1 and Figure 1. Similar to clinical intervention, the release of flow-diverter starts from the distal side of device to the proximal side of the device with respect to aneurysm sac. Figure 3 demonstrates snapshots of the LVIS EVO at three stages of deployment process in the WNBA I.

**Figure 4:**
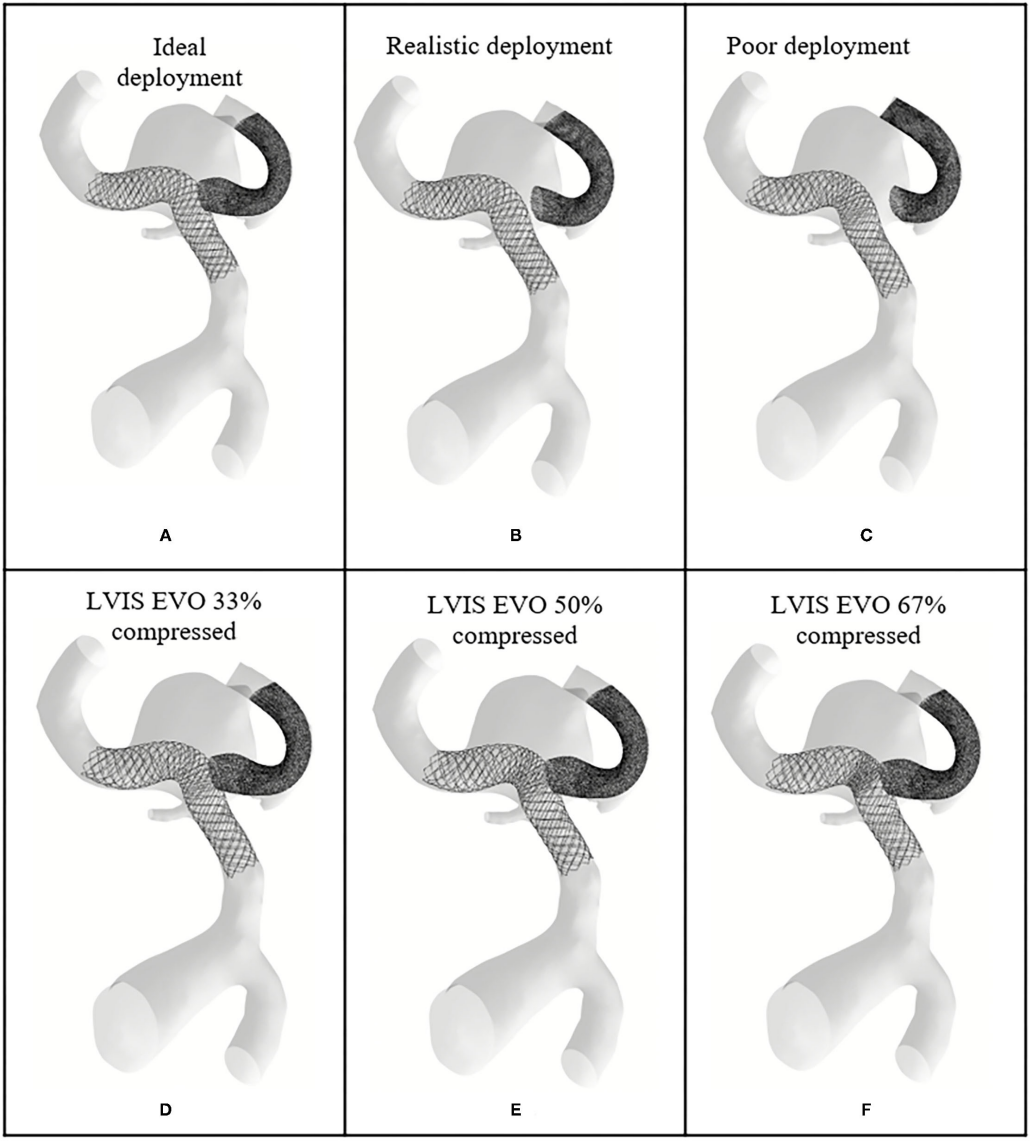
Deployed device positions in WNBA I. **(A)** Ideal deployment of LVIS EVO in the side daughter vessel. **(B)** Realistic deployment of LVIS EVO in the side daughter vessel. **(C)** Poor deployment of LVIS EVO in the side daughter vessel. **(D–F)** The deployment of LVIS EVO with 33, 50, 67% compression rate in the mid third part of the devices.

The virtual deployment is developed in Visual Studio 2019 and executed on a single 2.60 GHz core without parallelization such as multi-threading. The deployed position of each device for each geometry was achieved after around 50 iterations, and in a computation time of <1 min per case. The deployed devices are in a good contact with the vessel wall, with the wall considered as fixed throughout the deformation. Different deployment configurations of the SVB are achieved by editing the centerline of the secondary daughter vessel as shown in Figures 3A–C, 4A–C. The realistic deployment is validated with the post-intervention imaging, which give clear indication of device's position. Figures 3D–F, 4D–F shows the deployment of LVIS EVO and BABY LEO with different compression levels for the primary daughter vessel device (Stents II-IX). These configurations of deployed devices in each geometry are summarised in Tables 2, 3.

**Table 2:** Percentage reductions of total flow entering the sac of WNBA I with different deployment strategies and different compression level.

**WNBA I**			
**ID**	**Description**	**Qin (ml/min)**	**% Reduction**
ND	No devices	129.6	–
A	Real EVO (50%) and real SVB	46.32	64.3
B	Real EVO (50%) and ideal SVB	44.22	65.9
C	Real EVO (50%) and poor SVB	48.93	62.3
D	Uncompressed EVO (0%) and real SVB	67.9	47.6
E	Undercompressed EVO (33%) and real SVB	54.45	58.0
F	Overcompressed EVO (67%) and real SVB	46.35	64.2

**Table 3:** Percentage reductions of total flow entering the sac of WNBA II with different deployment strategies and different compression level.

**WNBA II**			
**ID**	**Description**	**Qin (ml/min)**	**% Reduction**
ND	No devices	78.18	–
A	Real Baby Leo (50%) and real SVB	22.93	70.7
B	Real Baby Leo (50%) and ideal SVB	23.87	69.5
C	Real Baby Leo (50%) and poor SVB	25.28	67.7
D	Uncompressed Baby Leo (0%) and real SVB	39.42	49.6
E	Undercompressed Baby Leo (33%) and real SVB	25.06	68.0
F	Overcompressed Baby Leo (67%) and real SVB	11.82	84.9

### Pre- and Post-intervention Haemodynamics

Calculations of inflow entering through the aneurysm neck in each case (WNBA I and II) are shown in Tables 2, 3 with values of 129.6 and 78.18 ml/min, respectively, representing ~100 and 60%, respectively, of the parent vessel (MCA) average flowrate. Velocity streamlines for both of these “No Device” cases are shown in the top rows of **Figures 6**, **7** below, where in both geometries relatively fast flow (~0.5 ms^−1^) can be seen to enter deep into the aneurysm dome. These jets of flow, most prominent in the WNBA I case, lead to regions of elevated WSS magnitude within the aneurysm dome caused by both the impact of the jet on the vessel wall (at the aneurysm tip) and the impingement flow leaving the dome (at the neck). The high aneurysm inflow rates (as a percentage of parent vessel flow rate), concentrated jet inflow, and regions of flow impingement have all been correlated with increased aneurysm rupture risk (32–34) and confirm the fragile nature of the aneurysm cases prior to clinical intervention.

**Figure 5:**
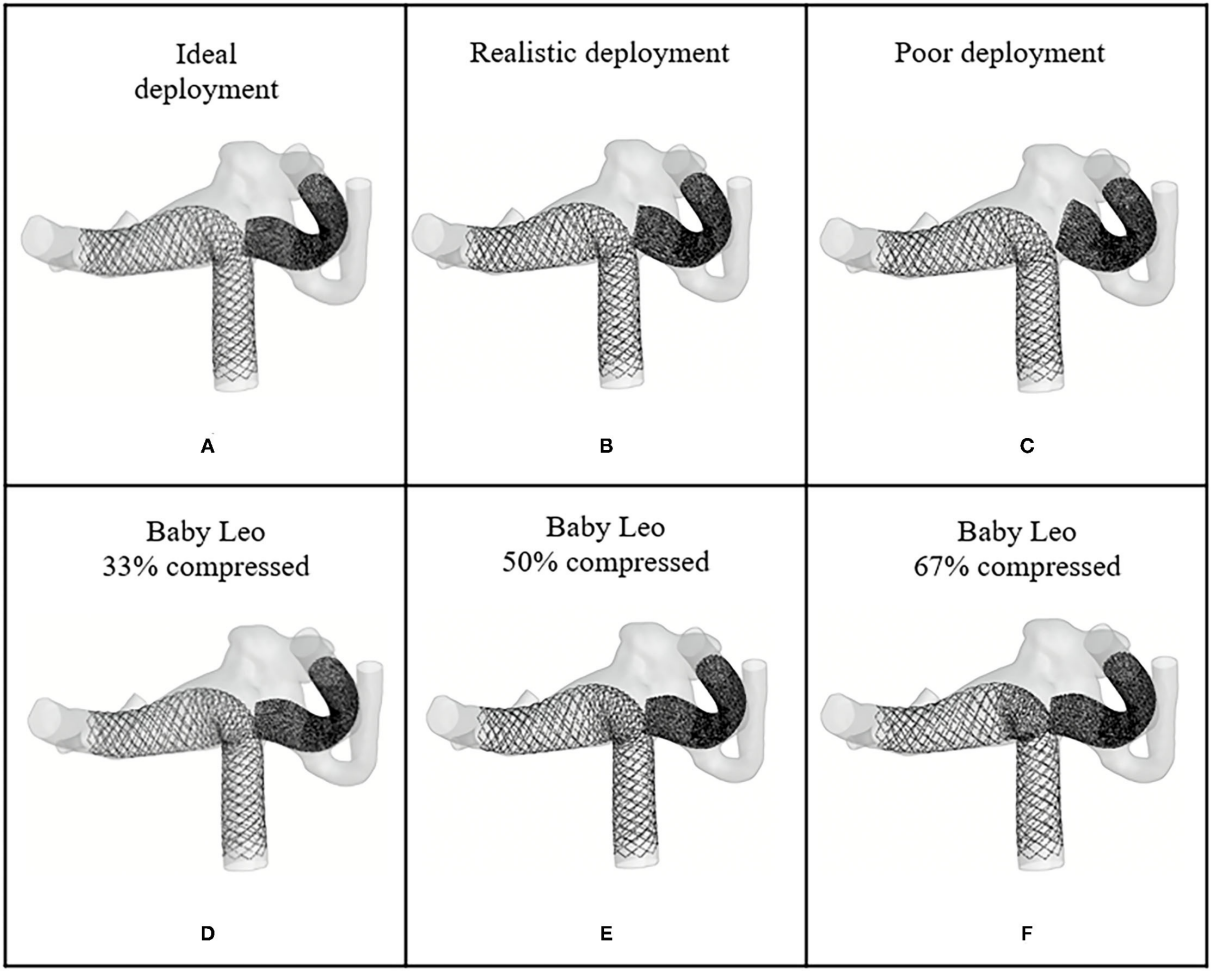
Deployed device positions in WNBA II. **(A)** Ideal deployment of SVB in the side daughter vessel. **(B)** Realistic deployment of SVB in the side daughter vessel. **(C)** Poor deployment of SVB in the side daughter vessel. **(D–F)** The deployment of BABY LEO with 33, 50, 67% compression rate in the mid third part of the devices.

**Figure 6:**
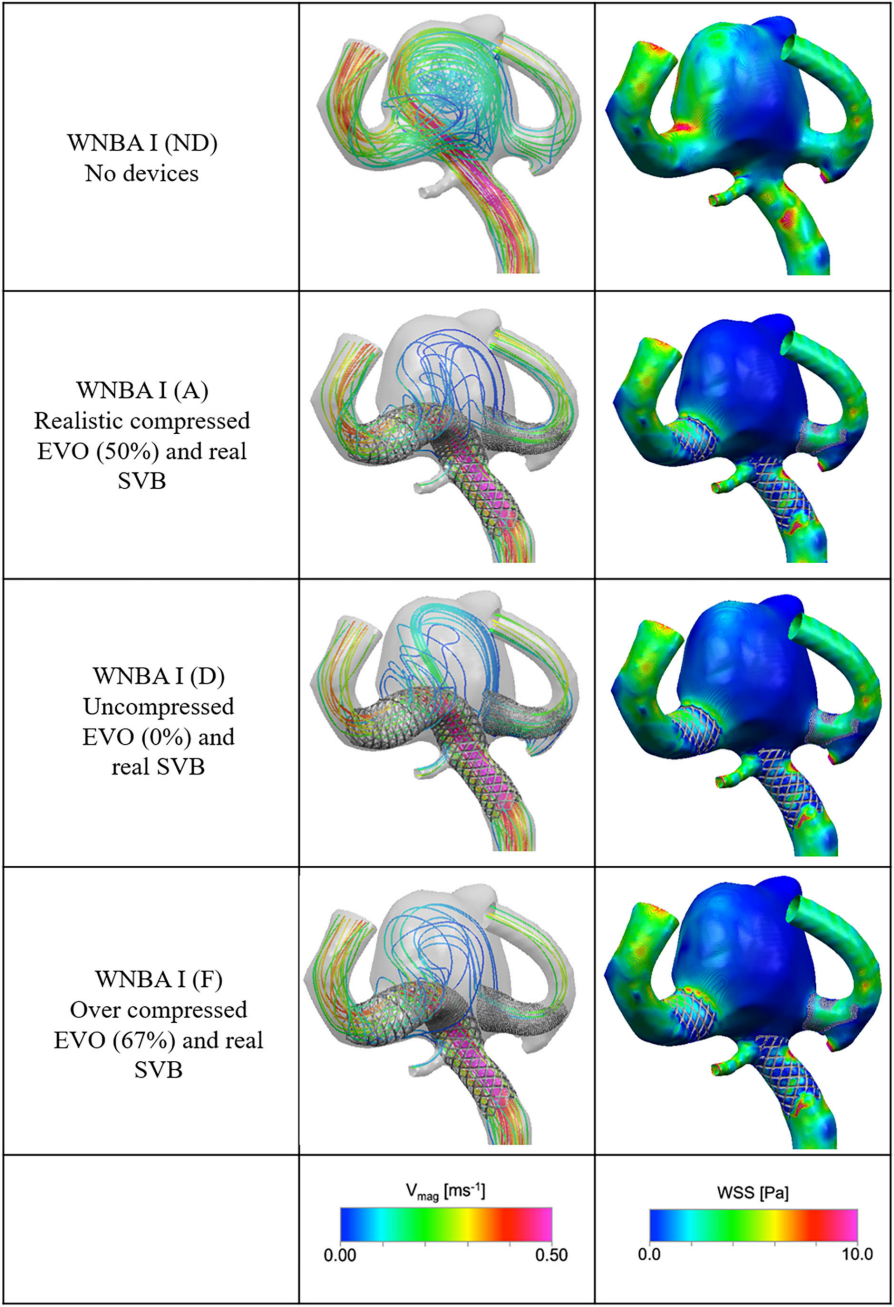
Velocity streamlines and WSS distributions for the No Device (ND) and selected device configurations (cases A, D, and F) for the WNBA I geometry.

In WNBA I the deployment of both devices in a realistic configuration (Case A in Table 2) can be seen to dramatically reduce the inflow into the aneurysm dome by 64.3%. Closer inspection of the second row of **Figure 6** shows that this reduction has been achieved by eliminating the inflow jet almost entirely (very little flow enters the left-hand portion of the aneurysm) and by substantially reducing the velocity of the flow that does enter aneurysm. Additionally, it is clear from the velocity streamlines of WNBA I A that the vast majority of flow entering the aneurysm dome then exits via the right-hand daughter vessel through the SVB device. This change in flow pattern has eliminated much of the flow impingement visible in the No Device case, where some flow exits the aneurysm dome via the left-hand daughter vessel causing elevated WSS (~8 Pa) at the aneurysm neck. The WSS magnitude plot for WNBA I A in Figure 5 confirms the elimination of the flow jet and impingement zone with the entire aneurysm dome WSS remaining around 2 Pa—a value typical of healthy vasculature.

Variation in the deployment position of the SVB device in WNBA I cases B and C shown in Table 2 indicate very little variation in inflow reduction with <5% difference in inflow reduction across the “real,” “ideal,” and “poor” configurations. This would suggest that the precise placement of the SVB device in the secondary daughter vessel does not substantially affect the overall flow-diverting effect of the Flow-T procedure in this geometry.

More variation in inflow reduction is seen by the level of compression of the LVIS EVO device in the WNBA I geometry as shown for cases D, E, and F in Table 2. With no compression of the EVO device (case D) the flow reduction drops by more than a quarter, compared to the realistic compression of 50%, to a flow reduction of 47.6%. The third row of Figure 6 shows the corresponding velocity and WSS distributions for this uncompressed case (D). While little difference in WSS distribution is visible between cases A and D, the lack of EVO compression in case D has resulted in a more substantial aneurysm inflow jet visible in the velocity streamline plots. The aneurysm flow pattern, with flow entering in the centre of the aneurysm neck, is similar in case D compared to case A, with the more open device pores in case D creating a single jet with velocity magnitudes around 0.25 ms-1. Ver or under-compressing the LVIS EVO device compared to the 50% length reduction used in the clinical deployment has a much more modest effect on inflow reduction as cases E and F in Table 2 illustrate. In these cases, the variation in inflow reduction of around 6% (or <10 ml/min) due to levels of compression appears to be dictated by the device pore size, and therefore the intensity of the aneurysm inflow jet. However, a slight shift in aneurysm flow pattern can be seen in the final row of Figure 6 whereby the higher degree of compression in the mid third of the device (67%) has resulted in not all of the aneurysm neck being covered by a compressed portion of the device, hence the reemergence of flow entering the left-hand portion of the neck where uncompressed EVO device is exposed.

**Figure 7:**
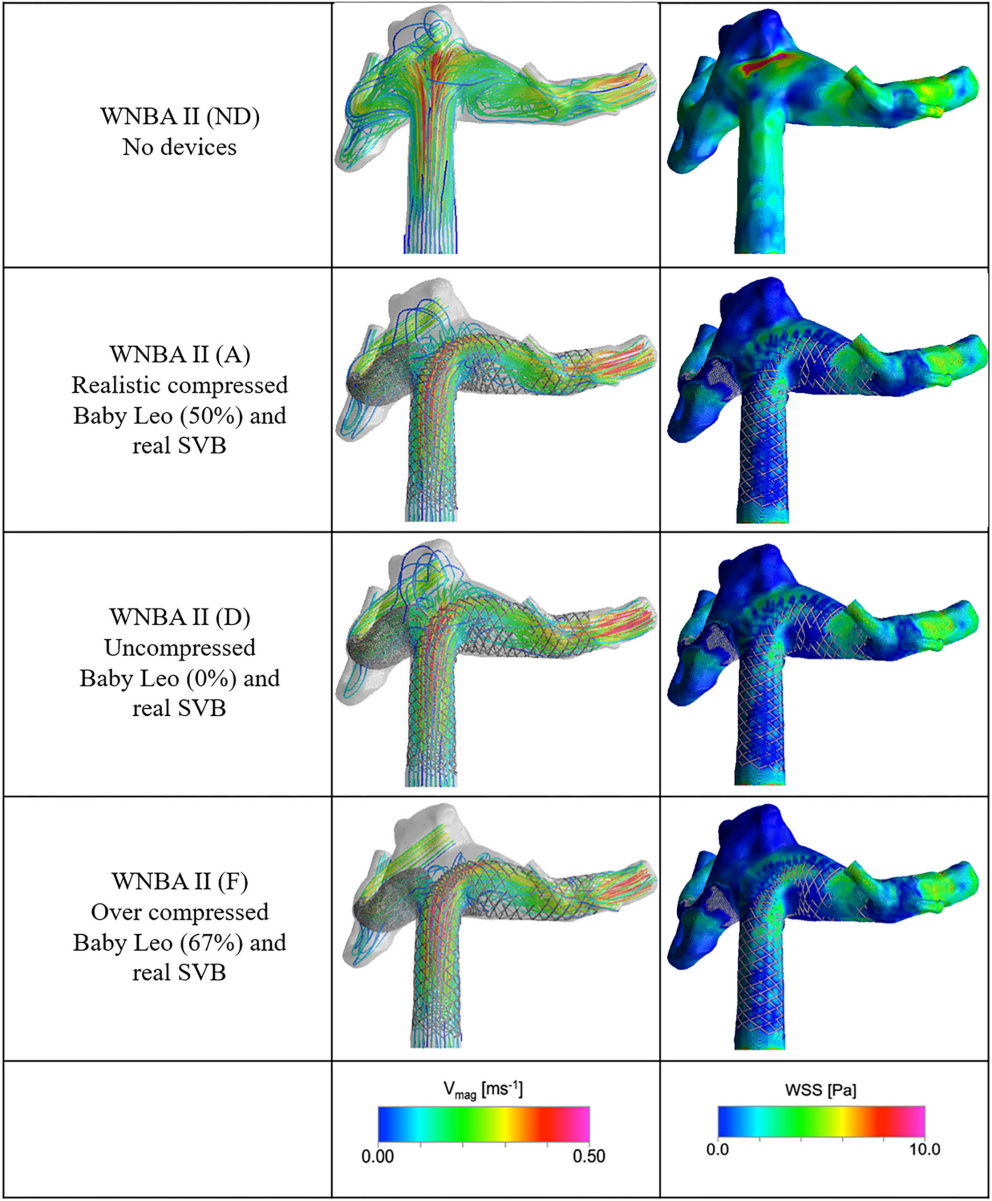
Velocity streamlines and WSS distributions for the No Device (ND) and selected device configurations (cases A, D, and F) for the WNBA II geometry.

There are similarities in the performance of the different configurations of devices for the WNBA II geometry as summarised in Table 3. Deployment of both devices in the realistic configuration (case A) results in an inflow reduction of 70.7%. Comparing the first and second rows of Figure 7, the result of the Flow-T intervention is similar to before: jets of fast flow (>0.5 ms^−1^) entering the aneurysm are reduced and the complex and impinged flow within the aneurysm dome is arrested to relatively simple circulating flow with blood entering the aneurysm on the right-hand side and exiting via the left. In particular, the concentrated region of high WSS magnitude (>8 Pa) seen close to the amorphous neck of the aneurysm in the No Device case is dramatically reduced following the device placements in all configurations (cases A–F). As before in the WNBA I case, the elimination of regions of elevated WSS and complex impinging flow would suggest that the Flow-T proceed substantially reduces the risk of aneurysm rupture and would promote thrombosis and aneurysm stabilisation.

Once again variation in positioning of the SVB device in the secondary daughter vessel in either the “poor,” “real,” or “ideal” configurations does little to affect the overall flow-diverting effect of the procedure. Less than 3% variation in inflow reduction is seen across the three cases (A–C) as shown in Table 3. Although it should be noted that the “ideal” SVB placement actually reduces the inflow reduction slightly when compared to the “real” position. This initially counter-intuitive effect results from the “ideal” SVB placement increasing the resistance to flow exiting the aneurysm dome compared to the “real” position, but the effect is small.

The variation in inflow reduction by Baby Leo device compression (cases D, E, and F in Table 3) is larger in the WNBA II geometry. A Flow-T configuration with compression applied to the Baby Leo stent results in an aneurysm inflow reduction of 49.6%—a figure similar to the first WNBA I geometry. Comparing the first and third row of Figure 7 it is clear that some flow complexity in the aneurysm dome remains when the Leo stent is uncompressed, but the magnitude of aneurysm flow velocity is substantially reduced compared to the No Device case. Finally, the over-compressed Baby Leo device in case F with 67% length reduction resulted in a very large increase in aneurysm inflow reduction to 84.9%. From the bottom row of Figure 7 it is clear that very little flow is entering the aneurysm dome in this case, and no coherent jet of flow is visible at all.

WNBA II is a complicated geometry to treat with any endovascular technique due to the extremely wide and amorphous aneurysm neck, which creates additional problems when defining the measurement plane through which aneurysm inflow can be measured. In the clinical approach coils were also added to the aneurysm sac prior to aid aneurysm isolation, although the similarity in modelled response of both WNBA I and WCNA II to Flow-T in this study suggest that such an adjunct measure may have not been necessary.

## Conclusions

This study detailed a computational workflow for virtually modelling device deployment configurations and simulating the resultant aneurysm haemodynamics for the novel Flow-T technique. The results obtained illustrate the value of using such tools to plan endovascular interventions and strategies, especially in complex aneurysm geometries and with a choice of device types and configurations—both key features of when the Flow-T technique would be chosen.

In this study, a fast deployment algorithm was used to deploy numbers of minimally invasive devices into the patient-specific geometries. The algorithms provide fast and precise deployment of devices which allows for the real time interaction and positioning optimization.

In the two patient-specific wide-necked aneurysms considered regardless of device configuration and compression aneurysm inflow is reduced by at least ~50% and regions of elevated WSS due to flow jetting and impingement are eliminated—all features associated with successful aneurysm isolation. These results reinforce the view that Flow-T represents a good endovascular option for hard-to-treat wide necked bifurcation aneurysms.

In the Flow-T approach the relative position of the secondary daughter vessel flow-diverter device (the SVB) was found to have a negligible effect on inflow reduction, aneurysm flow pattern, or WSS distribution in both aneurysm geometries. This suggests that the device placement in this vessel may be of secondary importance. By contrast, substantially more variation in inflow reduction and aneurysm flow pattern was seen due to variations in braided stent (LVIS EVO or Baby Leo) compression at the aneurysm neck. As such we conclude that the success of a Flow-T procedure is primarily dictated by the level of compression that the interventionalist applies to the braided stent. The similar positive results seen in both patient-specific geometries after virtually completing the Flow-T procedure suggest that the adjunct coiling that was utilised in the clinical approach to the second aneurysm geometry may have not been necessary for aneurysm stabilisation.

## Data Availability Statement

The original contributions presented in the study are included in the article/supplementary material, further inquiries can be directed to the corresponding author/s.

## Ethics Statement

Ethical review and approval was not required for the study on human participants in accordance with the local legislation and institutional requirements. The patients/participants provided their written informed consent to participate in this study. Written informed consent was obtained from the individual(s) for the publication of any potentially identifiable images or data included in this article.

## Author Contributions

ML virtual stenting algorithms development, literature research, manuscript preparation, and analysis statistical. TP CFD simulation and analysis CFD results and manuscript editing and preparation. YV manuscript editing and study design and data acquisition. PB clinical studies, provide essential data, and contribute in study concept and design. LM clinical studies and provide clinical data. All authors contributed to the article and approved the submitted version.

## Funding

This study was supported by the National Key R&D Program of China (2018AAA0102600).

## Conflict of Interest

The authors declare that the research was conducted in the absence of any commercial or financial relationships that could be construed as a potential conflict of interest.

## Publisher's Note

All claims expressed in this article are solely those of the authors and do not necessarily represent those of their affiliated organizations, or those of the publisher, the editors and the reviewers. Any product that may be evaluated in this article, or claim that may be made by its manufacturer, is not guaranteed or endorsed by the publisher.

## References

[1] Alfano JM Kolega J Natarajan SK Xiang J Paluch RA Levy EI. Intracranial aneurysms occur more frequently at bifurcation sites that typically experience higher hemodynamic stresses. Neurosurgery. (2013) 73:497–505. 10.1227/NEU.0000000000000016 23756745

[2] Zhang X-J Gao B-L Hao W-L Wu S-S Zhang D-H. Presence of anterior communicating artery aneurysm is associated with age, bifurcation angle, and vessel diameter. Stroke. (2018) 49:341–7. 10.1161/STROKEAHA.117.019701 29301972

[3] Ding YH Lewis D Kadirvel R Dai D Kallmes D. The woven endobridge: a new aneurysm occlusion device. AJNR Am J Neuroradiol. (2011) 32:607–11. 10.3174/ajnr.A2399 21330397 PMC8013102

[4] Mokin M Primiani CT Ren Z Piper K Fiorella DJ Rai AT. Stent-assisted coiling of cerebral aneurysms: multi-center analysis of radiographic and clinical outcomes in 659 patients. J Neurointerv Surg. (2020) 12:289–97. 10.1136/neurintsurg-2019-015182 31530655

[5] Akhunbay-Fudge CY Deniz K Tyagi AK Patankar T. Endovascular treatment of wide-necked intracranial aneurysms using the novel contour neurovascular system: a single-center safety and feasibility study. J Neurointerv Surg. (2020) 12:987–92. 10.1136/neurintsurg-2019-015628 31974281 PMC7509519

[6] Zhang Y Yang M Zhang H Zhang X Li Y Jiang C. Stent-assisted coiling may prevent the recurrence of very small ruptured intracranial aneurysms: a multicenter study. World Neurosurg. (2017) 100:22–9. 10.1016/j.wneu.2016.12.107 28062369

[7] Aydin K Men S Barburoglu M Sencer S Akpek S. Initial and long-term outcomes of complex bifurcation aneurysms treated by y-stent-assisted coiling with low-profile braided stents. Am J Neuroradiol. (2018) 39:2284–90. 10.3174/ajnr.A5869 30409852 PMC7655391

[8] Aydin K Sencer S Barburoglu M Berdikhojayev M Aras Y Sencer A. Midterm results of t-stent–assisted coiling of wide-necked and complex intracranial bifurcation aneurysms using low-profile stents. J Neurosurg. (2017) 127:1288–96. 10.3171/2016.9.JNS161909 28059656

[9] Pierot L Spelle L Cognard C Szikora I. Wide neck bifurcation aneurysms: what is the optimal endovascular treatment? J Neurointerv Surg. (2021) 13:e9. 10.1136/neurintsurg-2021-017459 33722965 PMC8053325

[10] Pierot L Biondi A. Endovascular techniques for the management of wide-neck intracranial bifurcation aneurysms: a critical review of the literature. J Neuroradiol. (2016) 43:167–75. 10.1016/j.neurad.2016.02.001 26976346

[11] Kara B Selcuk H Kilinc F Cakir C Zalov H. Combination of temporary bridging device (comaneci) and permanent stenting in the treatment of unruptured wide neck bifurcation aneurysms. Neuroradiology. (2021) 63:1–6. 10.1007/s00234-021-02677-z 33677621

[12] Cho Y Park S Lee J Seo J Kang H Kim J. Nonoverlappingy-configuration stenting technique with dual closed-cell stents in wide-neck basilar tip aneurysms. Clin Neurosurg. (2012) 70:244–9. 10.1227/NEU.0b013e31823bcdc5 21993186

[13] Makalanda H Wong K Bhogal P. Flow-t stenting with the silk vista baby and baby leo stents for bifurcation aneurysms - a novel endovascular technique. Interv Neuroradiol. (2020) 26:68–73. 10.1177/1591019919870618 31451027 PMC6998009

[14] Krupa K Brzegowy P Kucybała I Łasocha B Urbanik A Popiela JT. Endovascular embolization of wide-necked bifurcation aneurysms with the use of pCONus device: a systematic review and meta-analysis. Clin Imaging. (2021) 70:81–8. 10.1016/j.clinimag.2020.10.025 33130244

[15] Dandapat S Mendez-Ruiz A Martinez-Galdamez M Macho J Derakhshani S Foa Torres G. Review of current intracranial aneurysm flow diversion technology and clinical use. J Neurointerv Surg. (2021) 13:54–62. 10.1136/neurintsurg-2020-015877 32978269

[16] Srinivasan M Cherian J Levy EI Kan P. Chapter 22 - Endovascular flow diversion. In: Ringer AJ, editor. Intracranial Aneurysms. Academic Press (2018). p. 357–78.

[17] Zhu Y Zhan W Hamady M Xu XY. A pilot study of aortic hemodynamics before and after thoracic endovascular repair with a double-branched endograft. Med Novel Technol Dev. (2019) 4:100027. 10.1016/j.medntd.2020.100027

[18] Valent A Maier B Chabanne R Degos V Lapergue B Lukaszewicz AC. Anaesthesia and haemodynamic management of acute ischaemic stroke patients before, during and after endovascular therapy. Anaesth Crit Care Pain Med. (2020) 39:859–70. 10.1016/j.accpm.2020.05.020 33039657

[19] Zhang M Li Y Zhao X Verrelli DI Chong W Ohta M. Haemodynamic effects of stent diameter and compaction ratio on flow-diversion treatment of intracranial aneurysms: a numerical study of a successful and an unsuccessful case. J Biomech. (2017) 58:179–86. 10.1016/j.jbiomech.2017.05.001 28576622

[20] Chung B Cebral J. Cfd for evaluation and treatment planning of aneurysms: review of proposed clinical uses and their challenges. Ann Biomed Eng. (2014) 43:9. 10.1007/s10439-014-1093-6 25186432

[21] Spranger K Ventikos Y. Which spring is the best? Comparison of methods for virtual stenting. IEEE Transac Biomed Eng. (2014) 61:1998–2010. 10.1109/TBME.2014.2311856 24956618

[22] Peach TW Ngoepe M Spranger K Zajarias-Fainsod D Ventikos Y. Personalizing flow-diverter intervention for cerebral aneurysms: from computational hemodynamics to biochemical modeling. Int J Bibliogr Numer Methods Biomed Eng. (2014) 30:1387–407. 10.1002/cnm.2663 25045060

[23] *Manufacture Guidance: Leo+Leo+ Baby Self-Expandable Intracranial Stents*. (2021). Available online at: https://www.abmedica.org/en/solutions/neuroradiology/leo-and-leo-babyens/ (accessed June 01, 2021)

[24] van Doormaal JP Raithby GD. Enhancements of the simple method for predicting incompressible fluid flows. Num Heat Transfer. (1984) 7:147–63. 10.1080/01495728408961817

[25] Ni M-J Abdou MA. A bridge between projection methods and simple type methods for incompressible Navier–Stokes equations. Int J Numer Methods Eng. (2007) 72:1490–512. 10.1002/nme.2054

[26] Webster R. An algebraic multigrid solver for Navier-Stokes problems. Int J Numer Methods Fluids. (1994) 18:761–80. 10.1002/fld.1650180805

[27] Perktold K Peter R Resch M Langs G. Pulsatile non-Newtonian blood flow in three-dimensional carotid bifurcation models: a numerical study of flow phenomena under different bifurcation angles. J Biomed Eng. (1991) 13:507–15. 10.1016/0141-5425(91)90100-L 1770813

[28] Valencia A Solis F. Blood flow dynamics and arterial wall interaction in a saccular aneurysm model of the basilar artery. Comput Struct. (2006) 84:1326–37. 10.1016/j.compstruc.2006.03.008

[29] Dempere-Marco L Oubel E Castro M Putman C Frangi A Cebral J. Cfd analysis incorporating the influence of wall motion: application to intracranial aneurysms. In: Larsen R Nielsen M Sporring J, editors. Medical Image Computing and Computer-Assisted Intervention –MICCAI. 2006. Berlin; Heidelberg: Springer Berlin Heidelberg (2006). p. 438–45. 10.1007/11866763_54 17354802

[30] Peach TW Ricci D Ventikos Y. A virtual comparison of the eCLIPs device and conventional flow-diverters as treatment for cerebral bifurcation aneurysms. Cardiovasc Eng Tech. (2019) 10:508–19. 10.1007/s13239-019-00424-3 31286438 PMC6715664

[31] Peach TW Spranger K Ventikos Y. Virtual flow-diverter treatment planning: the effect of device placement on bifurcation aneurysm haemodynamics. Proc Inst Mech Eng H. (2017). 231:432–43. 10.1177/0954411916673674 27780870

[32] Hassan T Timofeev EV Saito T Shimizu H Ezura M Matsumoto Y. A proposed parent vessel geometry—based categorization of saccular intracranial aneurysms: computational flow dynamics analysis of the risk factors for lesion rupture. J Neurosurg. (2005) 103:662–80. 10.3171/jns.2005.103.4.0662 16266049

[33] Li W Wang Y. Regarding “differences in hemodynamics and rupture rate of aneurysms at the bifurcation of the basilar and internal carotid arteries. Am J Neuroradiol. (2017) 38:E51. 10.3174/ajnr.A5224 28522671 PMC7960420

[34] Castro M Putman C Sheridan M Cebral J. Hemodynamic patterns of anterior communicating artery aneurysms: a possible association with rupture. Am J Neuroradiol. (2009) 30:297–302. 10.3174/ajnr.A1323 19131411 PMC2735769

